# Benzothiophenone Derivatives Targeting Mutant Forms of Estrogen Receptor-α in Hormone-Resistant Breast Cancers

**DOI:** 10.3390/ijms19020579

**Published:** 2018-02-15

**Authors:** Kriti Singh, Ravi S. N. Munuganti, Nada Lallous, Kush Dalal, Ji Soo Yoon, Aishwariya Sharma, Takeshi Yamazaki, Artem Cherkasov, Paul S. Rennie

**Affiliations:** Vancouver Prostate Centre, University of British Columbia, 2660 Oak Street, Vancouver, BC V6H 3Z6, Canada; ksingh@prostatecentre.com (K.S.); rmunuganti@prostatecentre.com (R.S.N.M.); nlallous@prostatecentre.com (N.L.); kdalal@prostatecentre.com (K.D.); samanthayoon1993@gmail.com (J.S.Y.); rshar27@gmail.com (A.S.); takeshi.yamazaki@1qbit.com (T.Y.); acherkasov@prostatecentre.com (A.C.)

**Keywords:** breast cancer, estrogen receptor, hormone resistance, small molecule inhibitors, mutations, in silico modelling, activation function-2 (AF2) site

## Abstract

Estrogen receptor-α positive (ERα^+^) breast cancers represent 75% of all invasive breast cancer cases, while de novo or acquired resistance to ER-directed therapy is also on the rise. Numerous factors contribute to this phenomenon including the recently-reported *ESR1* gene mutations such as Y537S, which amplifies co-activator interactions with ERα and promotes constitutive activation of ERα function. Herein, we propose that direct targeting of the activation function-2 (AF2) site on ERα represents a promising alternative therapeutic strategy to overcome mutation-driven resistance in breast cancer. A systematic computer-guided drug discovery approach was employed to develop a potent ERα inhibitor that was extensively evaluated by a series of experiments to confirm its AF2-specific activity. We demonstrate that the developed small-molecule inhibitor effectively prevents ERα-coactivator interactions and exhibits a strong anti-proliferative effect against tamoxifen-resistant cells, as well as downregulates ERα-dependent genes and effectively diminishes the receptor binding to chromatin. Notably, the identified lead compound successfully inhibits known constitutively-active, resistance-associated mutant forms of ERα observed in clinical settings. Overall, this study reports the development of a novel class of ERα AF2 inhibitors, which have the potential to effectively inhibit ERα activity by a unique mechanism and to circumvent the issue of mutation-driven resistance in breast cancer.

## 1. Introduction

Breast cancer (BCa) is one of the most challenging oncologic problems and the second leading cause of cancer-related death in women. It has been estimated that the annual number of new cases of invasive BCa exceeds collectively over 280,000 in the USA and Canada [1]. It is a heterogeneous disease, the etiology of which appears to be related to a complex range of factors including age, race, lifestyle patterns, genetic and nutritional factors [2]. Preclinical and clinical investigations have demonstrated that estrogen receptor α-positive (ERα^+^) disease constitutes approximately 75% of all BCa cases and causes the majority of metastases and mortalities [3,4]. Since ERα (encoded by the *ESR1* gene) is the principal driver of BCa, agents that target the ERα signaling pathway such as aromatase inhibitors and ERα modulators are highly regarded as successful targeted therapies for pre- and post-menopausal patients [5,6]. However, despite the initial effectiveness of these drugs, intrinsic and acquired resistance remains a persistent problem that hampers the ultimate value of these treatments. As a consequence, surviving tumor cells progress to a hormone-resistant state [7,8].

Although diverse mechanisms of resistance to endocrine therapy have been described, recent evidence has identified acquired mutations in the *ESR1* gene, which confer ligand independent and constitutive receptor activation as a potential mechanism of resistance to the existing inhibitors [9,10,11]. These gene mutations were originally reported in a small cohort of metastatic BCa cases in 1997 [12]. In recent years, several independent groups performed studies utilizing the next-generation sequencing approach and reported that such mutations are present in ~20% of advanced, metastatic tumor samples previously treated with aromatase inhibitors [9,10,11]. Notably, these mutations occur rarely in primary BCa samples.

It should be emphasized that the most frequently-occurring *ESR1* mutations are located in the ligand binding domain (LBD) of ERα clustering around helix 12. Importantly, amino acids 534–538 frequently mutated in clinical samples are part of helix 12 and located in the proximity of the activation function-2 (AF2) area, a major protein-protein interaction site that recruits a variety of co-activators and mediates diverse functions of ERα [13,14,15]. It is estimated that such mutations can override the traditional ERα activation pathway and promote ERα function. A classic example is Y537S and D538G mutants that are constitutively active and promote increased interactions with co-activators at the AF2 site in an estrogen-independent fashion [16,17,18,19]. It has been reported that these mutants promote hormone-independent proliferation of tumor cell growth and reduce the efficacy of conventional drugs that target estrogen binding site (EBS) of the receptor.

The finding that activating mutations cluster in the LBD of ERα provides a tangible basis for the development of novel ERα targeting strategies. Hence, targeting the AF2 pocket of ERα bears the potential of not only inhibiting the wild-type ERα, but also its clinically-relevant LBD mutants. This strategy has been previously explored by other groups providing sufficient proof of the druggability of this site [20,21,22,23]. This study describes the structure-based optimization of our previously-reported AF2 inhibitor and experimental characterization of a further advanced and more potent AF2-directed small molecule that is effective against various ERα mutants.

## 2. Results

### 2.1. In Silico Identification and Experimental Evaluation of Benzothiophenone Analogues

**Figure 1:**
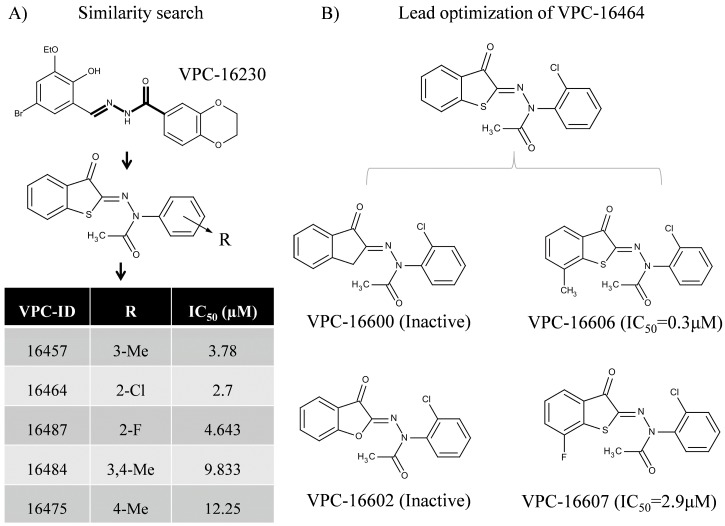
Identification and structure-guided lead optimization of benzothiophenone derivatives. (**A**) Previously-reported compound VPC-16230 (half maximal inhibitory concentration (IC_50_) 5.8 µM) used as a template to perform similarity search for more potent derivatives. The table shows IC_50_ values of the most promising leads observed against ERα in the luciferase reporter-based transcriptional assay. Among these, VPC-16464 (IC_50_ 2.7 µM) was identified as the most potent hit. (**B**) Lead optimization performed on VPC-16464. Different groups on the scaffold of VPC-16464 were tested. IC_50_ values in brackets are derived from the luciferase reporter-based transcriptional assay.

Previously, we reported on an AF2-specific inhibitor, Vancouver Prostate Centre-16230 (VPC-16230) that demonstrated promising ERα inhibition in estrogen-sensitive T47DKBluc cells and tamoxifen-resistant (TamR3) cells in vitro [24]. Herein, we used VPC-16230 as a chemical template (Figure 1A) to further identify improved AF2 inhibitors that exhibit enhanced target affinity and improved drug-like properties. A molecular similarity search was performed to identify analogues with different substitutions around the template structure. In particular, *Instant JChem*, a 2D similarity searching tool from ChemAxon [25], was employed to search through ZINC database v15 [26]. As a result, a total of 2000 compounds was selected for further analysis.

**Figure 2:**
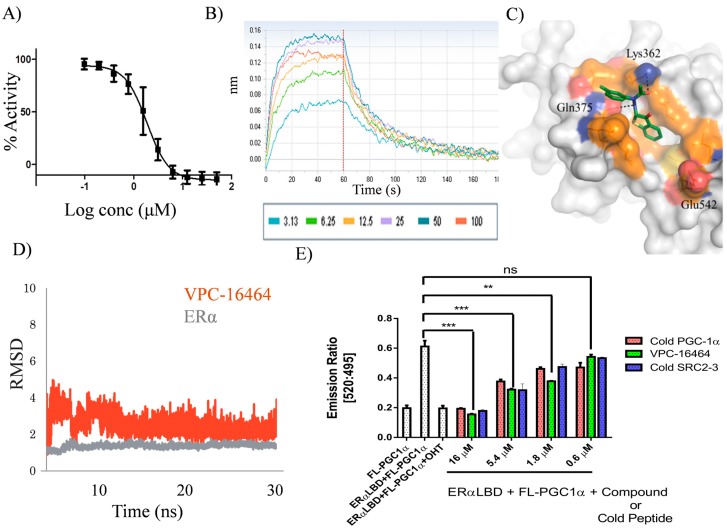
In silico predicted and experimental profile of VPC-16464. (**A**) Dose-response curve illustrating the inhibition effect (IC_50_ 2.7 μM) on ERα transcriptional activity in T47DKBluc cells using an estrogen-responsive luciferase reporter assay. Data points represent the mean of three independent experiments performed in triplicate and expressed as the percentage of luciferase activity relative to the 17 β-estradiol (E2) control. Error bars represent the standard error of mean (SEM) for 3 independent experiments performed in triplicates. Data were fitted using log_10_ of the concentration (conc) of the inhibitors vs. response with GraphPad Prism 6. (**B**) Biolayer interferometry (BLI) dose-response curves (3–100 μM) reflecting the direct binding of VPC-16464 to biotinylated ERα ligand binding domain (LBD) immobilized onto a streptavidin sensor. (**C**) The most stable conformation of VPC-16464 obtained from molecular dynamics (MD) simulations (green). H-bond interactions with the AF2 site residues are shown in black dotted lines. (**D**) RMSD curves demonstrating the individual stabilities of ERα and VPC-16464 upon complex formation during MD simulations. The orange curve shows that the ligand adopts a stable conformation in the AF2 pocket after 10 ns of simulation time. The grey curve shows that the ERα protein remains stable upon ligand binding. (**E**) VPC-16464 blocks the interactions between ERα-LBD and the LXXLL (L, leucine; X, any amino acid) motif containing fluorescein-peroxisome proliferator-activated receptor-γ coactivator 1α (FL-PGC1α) peptide in a dose-dependent manner as measured by the time-resolved fluorescence resonance energy transfer (TR-FRET) assay. 4-Hydroxytamoxifen (OHT) was used at a concentration of 1 µM. Error bars represent the SEM for 3 independent experiments performed in triplicates. *** *p* < 0.001, ** *p* < 0.01, unpaired *t*-test.

Using our in house computational drug discovery pipeline [24,27,28], those 2000 compounds were extensively evaluated in silico. Our previous study indicated that amino acids Lys362, Gln375, Val355, Ile358 and Leu379 are critical for protein-ligand coordination in the AF2 target site [24]. Therefore, the corresponding hydrophobic constraints were applied during in silico screening. Based on our previously-described computational protocols [24,27,28], five compounds belonging to the benzothiophenone series were identified from the set of 2000 candidates and then were purchased and tested for their ability to inhibit ERα activity using a luciferase reporter assay [24]. These compounds exhibited half maximal inhibitory concentration (IC_50_) values in the range of 2–13 µM (Figure 1A). Among those, a compound VPC-16464 demonstrated dose-dependent inhibition of ERα with a resulting IC_50_ of 2.7 µM (Figure 2A). Binding studies using biolayer interferometry (BLI) confirmed direct, reversible binding of VPC-16464 to ERα LBD in a dose-dependent manner (Figure 2B).

### 2.2. VPC-16464 Stably Binds to the AF2 Site during Molecular Dynamics Simulations

To gain a more detailed insight into interactions between AF2 site and VPC-16464, we conducted explicit solvent molecular dynamics (MD) simulations, where the binding pose of VPC-16464 predicted by the Glide SP program [29] was used as a starting point.

During 30-nanoseconds (ns) MD simulations, it was observed that the compound was tightly bound to the target AF2 site throughout the simulation period. Based on generalized Born (GB) model augmented with the hydrophobic solvent accessible surface area (SA) (GBSA) binding free energy calculations [30], the most stable conformation of VPC-16464 was extracted to perform further lead optimizations (Figure 2C). The individual stabilities of the receptor and ligand conformations in the ligand-receptor complex were confirmed by calculating the root mean square deviation (RMSD) between their initial conformations and snapshots of each of them during the entire simulation time. RMSD values are helpful in estimating the deviation of atoms in 3D space from their original position. Figure 2D shows that both ligand and protein exhibit consistent RMSD values. The orange curve depicts that the ligand adopts a stable conformation in the AF2 pocket from 10 ns onwards (with an average RMSD ~3Å), clearly suggesting that the ligand fits well in the pocket. The grey curve indicates that the ERα protein remains very stable upon ligand binding and throughout the simulation time. Moreover, binding of the ligand does not alter its conformation.

Using the frequent contacts map, we observed that AF2 residues Val355, Ile358, Lys362, Phe367, Gln375 and Leu379 interact with VPC-16464 for over 60% of the total MD simulation time. Importantly, the benzothiophenone moiety forms strong H-bond interactions with Lys362 and Gln375 (Figure 2C), whereas Ile358, Phe367, Val368 and Leu379 residues make strong hydrophobic contacts with the chemical core.

Since MD simulations suggested that VPC-16464 is a stable AF2 binder, the time-resolved fluorescence resonance energy transfer (TR-FRET) assay was performed to evaluate its ability to displace co-activator from the AF2 site. In this assay, a three-fold dilution range of VPC-16464 was utilized. As anticipated, VPC-16464 successfully displaced the fluorescein-Peroxisome proliferator-activated receptor-γ coactivator 1α (FL-PGC1α) peptide in a dose-dependent manner. This response was similar to the control peptides (Figure 2E).

### 2.3. Lead Optimization of VPC-16464

Since VPC-16464 demonstrated promising anti-ERα potency and could effectively displace peptide from the AF2 site, structure-guided medicinal chemistry (med chem) optimization was performed on the compound using the most stable binding pose of VPC-16464. The initial two derivatives, VPC-16600 and VPC-16602, were designed by replacing S with C and O atoms, respectively. These modifications abolished the cellular activity of these derivatives and their binding to the ERα (Figure 1B) and provided important structure-activity relationship (SAR) insight into protein-ligand coordination, such as the importance of non-polar interactions.

**Figure 3:**
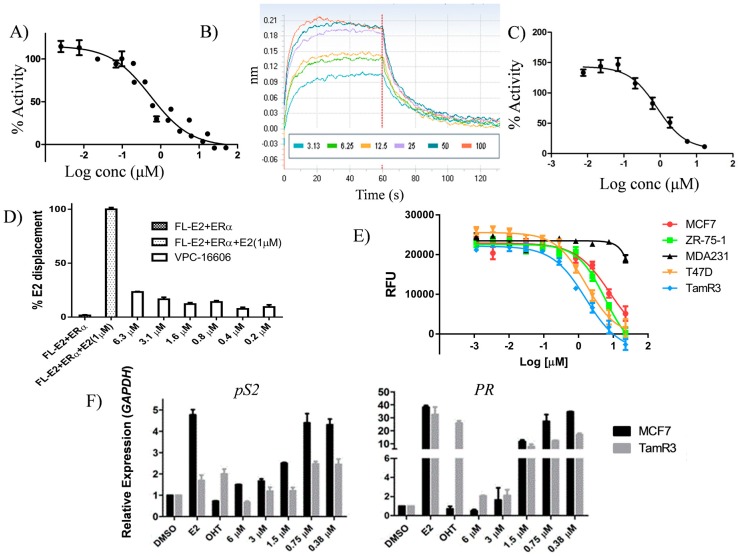

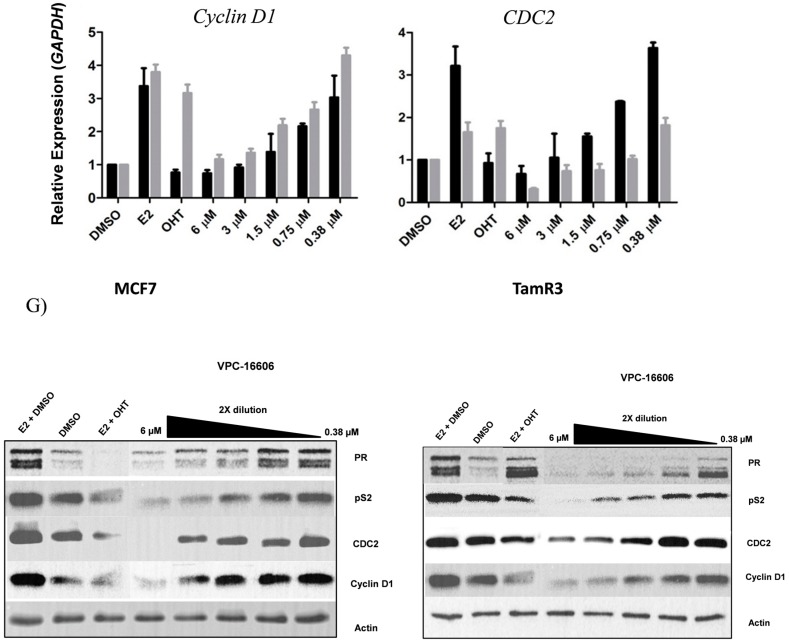
Activity profile of VPC-16606. (**A**) Dose-response curve illustrating the inhibitory effect of VPC-16606 (IC_50_ 0.3 μM) on ERα transcriptional activity in T47DKBluc cells. (**B**) BLI dose-response curves (3–100 μM) reflecting the direct binding of VPC-16606 to ERα LBD. (**C**) Mammalian two-hybrid assay in MDA-MB-231 cells transfected with pACT-ERα-LBD, pBIND-Steroid receptor coactivator protein-3 (pBIND-SRC-3), pG5luc and a constitutively-active Renilla reporter. Cells were treated with VPC-16606 in the presence of 1 nM E2. The compound prevented the interaction between ERα-LBD and SRC-3 fusion proteins in a dose-dependent manner (IC_50_ 0.8 μM), suggesting its AF2-mediated mode of action. (**D**) E2 displacement was measured by the fluorescence polarization assay to eliminate the possibility that VPC-16606 binds to the estrogen binding site (EBS). VPC-16606 showed minimal displacement of fluorescein labelled E2 (FL-E2) from the EBS compared to the positive control (E2) at the highest concentration (6 µM). (**E**) Dose-response curves of VPC-16606 showing the decrease in growth of ERα^+^ cell lines as assessed by the Presto Blue viability assay. (**F**) VPC-16606 decreased mRNA levels of ERα-dependent genes, *pS2*, *PR*, *Cyclin D1* and *CDC2* in MCF7 and tamoxifen-resistant (TamR3) cells. Cells were treated with the test compound for 24 h in the presence of 1 nM E2. OHT (1 µM) was used as the control. (**G**) Western blots showing decreased expression of pS2, PR, Cyclin D1 and CDC2 proteins in MCF7 and TamR3 cell lines upon 24 h of treatment with VPC-16606. Error bars on all graphs indicate standard error of the mean (SEM) for 3 independent experiments performed in triplicates.

Another focal point of the study was the evaluation of the effect of substitutions at the benzothiophenone moiety of VPC-16464. As illustrated in Figure 2C, the ligand is buried deeply into the AF2 cavity. Hence, the addition of a hydrophobic group should enhance non-polar interactions with neighboring AF2 residues and contribute towards improved ligand binding. Therefore, derivatives VPC-16606 and VPC-16607 were designed by adding methyl and fluorine at the seventh position of the benzothiophenone core. As anticipated, VPC-16606 demonstrated a considerable increase in potency (IC_50_ 0.3 μM as shown in Figure 3A) due to stronger van der Waals contacts with Val355, Ile358 and Leu359. Moreover, the direct reversible interaction between VPC-16606 and the ERα LBD was also detected by the BLI assay (Figure 3B).

### 2.4. VPC-16606 Blocks the Interactions between Co-Activators at the ERα AF2 Site

The direct effect of VPC-16606 on ERα-co-activator recruitment was assessed by the mammalian two-hybrid system (Promega, Madison, WI, USA). MDA-MB-231 cells were transfected with pACT-ERα-LBD, pBIND-Steroid receptor coactivator protein-3 (pBIND-SRC-3), a luciferase reporter plasmid containing a GAL4 recognition sequence and a constitutively-active Renilla reporter plasmid. The cells were treated with a three-fold dilution of VPC-16606 starting at 50 µM. The compound significantly inhibited the interaction between ERα and SRC-3 in a dose-dependent manner (Figure 3C). This provides direct evidence that the compound shows AF2-mediated activity.

It should be noted that selective estrogen receptor modulators (SERMs) such as tamoxifen displace co-activator protein from AF2 allosterically, i.e., by binding to the estrogen binding site (EBS). To ascertain whether VPC-16606 is not an EBS binder, 17 β-estradiol (E2) displacement was assessed with the Polar Screen Estrogen Receptor-α Competitor Green Assay Kit (P2698, Life Technologies, Carlsbad, CA, USA). The compound showed minimal displacement of fluorescein labelled E2 (FL-E2) from the EBS even at a concentration 10-fold higher than its IC_50_ (Figure 3D), suggesting that it is highly unlikely that the effects observed are through its activity at EBS and that it is a true AF2 binder.

### 2.5. VPC-16606 Inhibits ERα-Dependent Cell Growth and Gene Expression

The Presto Blue cell viability assay was used to assess the growth inhibitory potential of VPC-16606 on ERα^+^ T47D, MCF7, ZR75-1 and TamR3 cells. They were treated for 96 h with a two-fold dilution range of the compound starting at 50 µM. As featured in Figure 3E, the compound could significantly reduce the growth of ERα^+^ cell lines without any effect on ERα^−^ MDA-MB-231 BCa cells, which suggests that the compound exhibits an ERα-mediated mode of action. Moreover, it was observed that VPC-16606 significantly downregulates the expression levels of ERα-dependent genes such as *pS2*, *PR*, *Cyclin D1* and *CDC2* at the mRNA level in MCF7 and TamR3 cells (Figure 3F). However, at the protein level, this effect was observed at higher doses for the same duration of treatment (Figure 3G).

### 2.6. VPC-16606 Diminishes ERα Binding on Estrogen Response Elements (ERE)

Binding of co-activators to the AF2 site is crucial for the formation of the transcription complex. The p160 family of co-activators, such as SRC-1–3, aid the transcription process by interacting with hormone-bound ERα to facilitate further recruitment of other components of the transcriptional complex. They do so by interacting with the histone acetyltransferases CREB-binding protein (CBP) and p300 through their activation domain 1 (AD1), with the histone methyltransferases coactivator associated arginine methyltransferase 1 (CARM1) and protein arginine methyltransferase 1 (PRMT1) through their AD2 and with switch/sucrose non-fermentable (SWI/SNF) (an ATP-dependent chromatin remodeling complex) through their AD3. The formation of this co-activator complex results in chromatin remodeling and bridges the hormone-activated ERα with the general transcription machinery for transcriptional activation of its specific target genes [31]. Thus, the binding of co-activators to ERα represents the first crucial step in the initiation of transcription. Since the developed ERα inhibitors are designed to inhibit co-activator recruitment, it was important to evaluate whether this would affect ERα binding to the EREs of estrogen-regulated genes.

**Figure 4:**
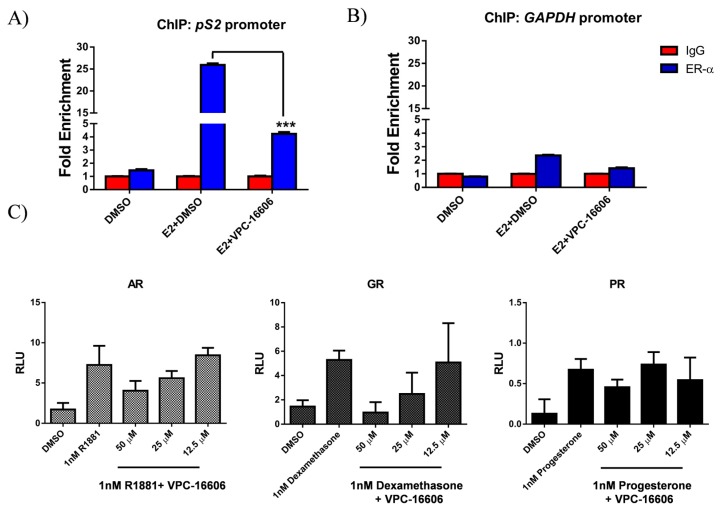
Further activity profile of VPC-16606. (**A**,**B**) Chromatin immunoprecipitation (ChIP) analysis of ERα binding to the *pS2* enhancer, or the *GAPDH* promoter, in MCF7 cells. Where indicated, ERα was stimulated with 1 nM E2 or dimethyl sulfoxide (DMSO) alone, and the compound was administered at a 6 μM concentration. Sheared chromatin-protein complexes were precipitated with the ERα antibody, reverse cross-linked and analyzed by quantitative PCR. The results are normalized as fold enrichment over precipitation with a rabbit isotype control IgG antibody for each condition tested. Error bars indicate standard error of the mean (SEM) for 3 independent experiments performed in triplicates. Fold enrichment from the compound tested condition (ERα antibody) was statistically compared against DMSO + E2 with a two-tailed *t*-test (unpaired) *** indicates *p*-value <0.001. (**C**) Specificity of VPC-16606 was evaluated in the luciferase reporter assay. PC3 cells were transfected with plasmids expressing androgen receptor (AR), progesterone receptor (PR) or glucocorticoid receptor (GR) along with their responsive luciferase reporter constructs bearing androgen response element (ARE), progesterone response element (PRE) or glucocorticoid response element (GRE), respectively. The cells were treated for 24 h followed by lysis and measurement of luciferase signal. Y-axis represents relative luminescence (RLU) after normalizing to constitutive renilla reporter.

To test this hypothesis, we performed chromatin immunoprecipitation (ChIP) assays in MCF7 cells. The cells were treated for 24 h with vehicle and E2 either alone or in combination with VPC-16606. The analysis of chromatin demonstrated that VPC-16606 significantly reduced ERα pull down of the promoter of the ERα-regulated gene, *pS2*, compared to E2 treatment alone (Figure 4A). Control experiments revealed no pull down of the enhancers in the absence of E2, under any condition with the *GAPDH* promoter negative control (Figure 4B). These results provide an explanation for the transcriptional inhibition of ERα observed in the luciferase and qRT-PCR analyses. Thus, from these results, we show that blocking co-activator recruitment at the AF2 site destabilizes binding of ERα on ERE and possibly prevents assembly of the transcription complex.

### 2.7. VPC-16606 is Selective towards ERα

The selectivity of VPC-16606 towards ERα was tested using a luciferase assay. Figure 4C shows that VPC-16606 does not affect the activity of androgen receptor (AR), glucocorticoid receptor (GR) and progesterone receptor (PR) even at high doses of up to 25 µM compared to their respective stimulated controls. At 50 µM, however, we see some effect, but this could be because the compound affects cell viability at this concentration as seen in the growth assay on PC3 cells (Figure S1). Moreover, this concentration is very high compared to the ones used in all our assays.

### 2.8. VPC-16606 Inhibits Clinically-Relevant Mutant Forms of ERα

Mutations in the *ESR1* gene have long been linked to endocrine therapy resistance. Recently, with the advancement of sequencing techniques, recurrent mutations on *ESR1* have been found to occur more frequently than expected in patients with ERα^+^ metastatic disease and contribute to acquired endocrine therapy resistance [9,10,11]. The Y537S mutant has been shown by different groups to be constitutively active. The location of this residue outside the AF2 pocket implies that the efficacy of VPC-16606 should not be affected by this mutation.

**Figure 5:**
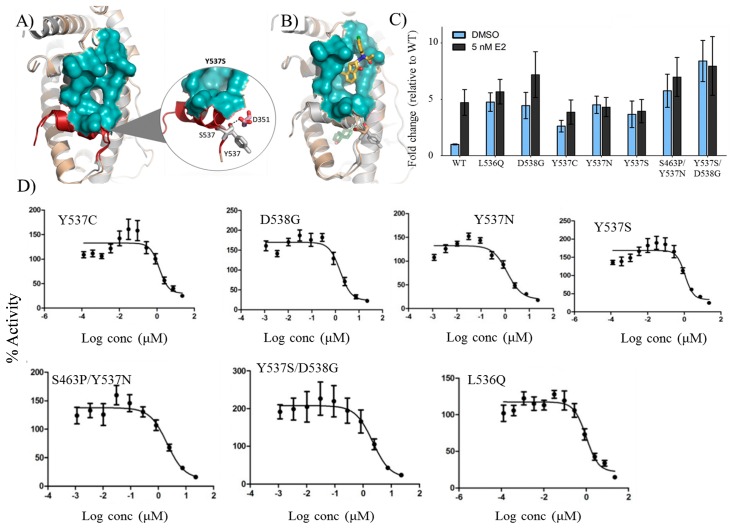
Predicted MD simulations and experimental effect of VPC-16606 on mutant forms of ERα. (**A**) Overlapping MD simulated structures of wild-type (WT) (grey) and Y537S (beige) forms of ERα show that an additional hydrogen bond formed between S537 and D351 in the mutant ERα (shown in a circle) causes the AF2 pocket (cyan) to be constitutively open. (**B**) Binding pose of VPC-16606 (yellow) in WT and mutant forms overlapped with each other shows that the compound would bind in a similar manner in both forms of ERα. (**C**) E2 responsiveness of the synthesized mutants. (**D**) pcDNA3.1 plasmid encoding either the full-length WT or mutant forms of ERα was transfected into MDA-MB-231 cells along with the 3X-ERE-TATA luciferase reporter plasmid. The cells were treated with a two-fold dilution of VPC-16606 starting at 50 µM. The compound successfully inhibited the constitutively-active mutant forms of the receptor in a dose-dependent manner. Data points are expressed as the percentage of luciferase activity relative to DMSO control. Data were fitted using the log_10_ of concentration of the inhibitors vs. the response with GraphPad Prism 6. Error bars indicate the standard error of the mean (SEM) for 3 independent experiments performed in triplicates.

In order to study the structural differences between Y537S and the wild-type (WT) receptor to decipher the molecular mechanisms responsible for the hormone-independent activities of the mutant form, an MD study was conducted. Computational modeling on the Y537S form (in the absence of E2) revealed that the mutation Y537S, located on helix 12 (H12), facilitates an additional H-bond interaction with the neighboring D351 residue (Figure 5A). This causes the receptor to be in a constitutively-open conformation, resulting in recruitment of co-activators independent of E2 activation. It should be noted that this particular scenario does not occur in WT ERα (Figure 5A).

Next, the effect of VPC-16606 binding on both WT and Y537S forms was investigated. It was observed that there is no difference in compound orientation as the topology and the features of the AF2 site in both mutant and WT forms are highly similar (Figure 5B). Moreover, the free energies of binding for WT ERα-16606 and Y537S ERα-16606 complexes are similar. Based on these observations, it was anticipated that VPC-16606 should exert similar activity on these two forms of ERα.

To test this hypothesis, a site-directed mutagenesis study was initiated on WT-ERα. MDA-MB-231 cells that were transfected with plasmids encoding either WT or mutant ERα (L536Q, Y537S, Y537C, Y537N, D538G, Y537S/D538G, S463P/Y537N) along with an E2-responsive luciferase reporter plasmid and a constitutively-active Renilla reporter plasmid. The cells were treated with VPC-16606 with two-fold dilution starting at 50 µM. As shown in Figure 5C, these mutants were constitutively active and, unlike WT, showed stimulation independent of E2 treatment. The compound successfully inhibited all the mutant forms with IC_50_ values in the range of 0.5–1 µM (Figure 5D). This is particularly important because they corroborate the idea that such clinically-relevant mutant forms of the receptor, which cause the receptor to be E2 independent, can be inhibited by targeting an alternative functional site on the receptor.

## 3. Discussion

A systematic computer-guided optimization was performed on a series of benzothiophenone derivatives as highlighted by Figure 1. The identified molecule, VPC-16464, exhibited an IC_50_ value of 2.7 µM in a luciferase-based transcriptional assay and effectively displaced co-activator peptides from the AF2 site in an in vitro TR-FRET assay. The most stable conformation of VPC-16464 identified using MD simulation analysis (Figure 2) enabled us to perform structure-based lead optimization to enhance the potency of the benzothiophenone chemicals as illustrated in Figure 1. Replacing the S group of VPC-16464 with either C or O completely obliterated its activity, highlighting its importance in compound binding. Adding fluorine to the benzothiophenone core did not alter the potency since fluorine does not facilitate non-polar interactions, whereas the addition of a methyl group resulted in a substantial improvement in potency (VPC-16606) with an IC_50_ of 0.3 µM. Computational modeling of the ERα AF2-VPC-16606 complex revealed that the presence of a methyl group in the ligand results in the formation of additional van der Waals interactions with neighboring AF2 residues, which may contribute to the improvement in potency.

It was further established that VPC-16606 did not displace E2 from the EBS, but rather blocked the interaction with the well-known p160 family of ERα co-activators, particularly SRC-3, as measured by the mammalian two-hybrid assay, thereby confirming the AF2-specific mechanism of ERα inhibition (Figure 3A–D). This compound exhibited a dose-dependent anti-proliferative effect against a panel of ERα^+^ cell lines (T47D, MCF7 and ZR-75-1) with no effect on ERα^−^ MDA-MB-231 cells, confirming its ERα-mediated activity and ruling out cytotoxic effects (Figure 3E). Inhibition of activity by blocking co-activator recruitment resulted in downregulation of well-known ERE-driven genes and others regulated by tethered ERα interactions (Figure 3F). The downregulation of *CDC2* and *Cyclin D1*, in particular, could explain the observed growth inhibition of ERα^+^ cells. Downregulation of these genes in MCF7 could be recapitulated after 24 h of compound treatment, but the corresponding effect on protein levels of *CDC2* and *Cyclin D1* were not observed (Figure 3G) compared to TamR3, suggesting residual proteins may be responsible for the differential growth effects in these cell lines. The presence of residual proteins could partly explain the differential response to compounds in luciferase assays versus growth assays. Cell growth is regulated by multiple factors beyond ERα; therefore, a higher dose of compound may be required to manifest growth inhibition compared to transcriptional reporter assays, which reflect the direct interaction between the compound and ERα. Despite these concerns, it is promising that our AF2 inhibitor demonstrated significant anti-proliferative effects on TamR3 cells given the ultimate goal of targeting endocrine and tamoxifen-resistant BCa.

Following ChIP analysis in MCF7 cells, it was demonstrated that targeting the AF2 site with VPC-16606 destabilizes binding of ERα on the ERE of the *pS2* promoter (Figure 4A) in addition to preventing co-activator recruitment required for ERα transcriptional activity (Figure 3C). Collectively, these data suggest a plausible mechanism of action of the compound and provide scope for further investigation. ChIP-seq analysis in the presence and absence of compound would provide a more detailed view of the global effect on chromatin binding. Moreover, it would be interesting to look at the compound’s effect on interaction between ERα and chromatin-bound proteins like c-Jun and activating transcription factor-2 (ATF-2) that are involved in tethered interactions at promoters of genes like *Cyclin D1* [32]. Whether the phosphorylation status of ERα affects compound activity was not assessed in this study. However, from our MD simulations, we observed that the compound does not interact with the residues that are known to be phosphorylated; hence, we do not anticipate any effect on compound binding or nuclear localization of ERα.

VPC-16606 treatment resulted in peptide displacement when tested against the purified ERβ LBD (Figure S2). This result is not surprising considering the sequence similarity between both ER isoforms and the fact that tamoxifen also binds to ERβ [33]. Given that tamoxifen acts as a pure antagonist of ERβ [34], the effect of VPC-16606 on ERβ and its corresponding implications in breast cancer will also need to be evaluated. Moreover, the role of ERβ in breast cancer biology is not yet clear [35], with some evidence pointing towards ERβ exerting proliferating effects in the absence of ERα [36].

Recently, Toy et al. and Robinson et al. performed two independent studies, and both groups identified recurrent mutations in *ESR1* gene that were not observed in untreated populations [10,11]. They confirmed that ERα mutants are constitutively active (even in the absence of its natural ligand, E2) and promote hormone-independent tumor growth after estrogen deprivation in vivo. In our MD stimulations, the mutant form adopts an agonist conformation similar to E2 bound WT ERα. An additional H-bond formed between S537 and D351 stabilizes the AF2 site formation irrespective of prior E2 binding. Consequently, AF2 is capable of recruiting coactivator proteins, continuously leading to constitutive activity of mutant forms (as observed in Figure 5A).

In such clinical scenarios, the relevance of our AF2 inhibitor is the effectiveness against known ERα mutants that cause resistance to current therapies. When VPC-16606 was tested against five single and two double constitutively-active mutant forms of ERα, it successfully inhibited the transcriptional activity of these mutants with similar efficacy as against the WT receptor (0.5–1 µM; Figure 5D). Notably, sub-micromolar IC_50_ values offer an advantage over conventional hormone therapies, which require significantly higher doses. Caution should be exercised with this approach, however, since dual agonist/antagonist activity of SERMs increases the risk of developing endometrial cancer.

The authors would like to add that although we have observed promising results in vitro, especially on the mutant forms of ER, anti-tumor effects of compound-treatment in animal models of BCa remain to be explored. Systemic effects on other ERα-sensitive tissues and pharmacokinetic and pharmacodynamic properties must be evaluated to achieve that goal, but this is beyond the scope of this study.

## 4. Materials and Methods

### 4.1. In Silico Modeling of AF2 Inhibitors

MD simulations of VPC-16464, ERα WT and ERα Y537S were performed as described previously [37].

### 4.2. Cell Culture

T47DKBluc cells (ATCC^®^ CRL-2865^TM^, Manassa, VA, USA) stably transfected with a 3X-ERE–promoter–luciferase reporter gene construct, pGL2.TATA.Inr.luc.neo [38], were used to measure inhibition of ERα-driven transcriptional activity of the luciferase reporter.

MDA-MB-231, PC3, MCF7, T47DKBluc and ZR75-1 cell lines were obtained from ATCC. Tamoxifen-resistant MCF7-derived cell line, TamR3, was obtained from Euphemia Leung (University of Auckland, New Zealand). Cells were cultured at 37 °C in a humidified incubator with 5% CO_2_. The cell lines were maintained in the following culture media: T47DKBluc: phenol red–free RPMI1640 (Gibco, Life Technologies, Carlsbad, CA, USA) containing 4.5 g/L glucose (Sigma-Aldrich, St. Louis, MI, USA), 10 mM 4-(2-hydroxyethyl)piperazine-1-ethanesulfonic acid (Sigma-Aldrich), pH 7.5, 1 mM sodium pyruvate (Life Technologies, Carlsbad, CA, USA), 0.2 U/mL insulin (Sigma-Aldrich) and 10% FBS; MCF7 and ZR75-1: phenol-red-free RPMI 1640 supplemented with 10% fetal bovine serum (FBS); MDA-MB-231 and PC3: Dulbecco’s Modified Eagles Medium (DMEM) (Hyclone, Thermo Fisher Scientific, Waltham, MA, USA) supplemented with 10% FBS. TamR3: phenol red-free RPMI 1640 containing 10% charcoal-stripped serum (CSS) and 1 μM tamoxifen.

### 4.3. Chemicals and Antibodies

17 β-estradiol (E2), tamoxifen and 4-Hydroxytamoxifen (OHT) were obtained from Sigma-Aldrich. Anti-progesterone receptor and Cyclin D1 antibodies were purchased from Abcam (Cambridge, UK). Mouse monoclonal anti-ERα antibody (6F11) was obtained from Leica (Wetzlar, Germany). All other chemicals have been described previously [24].

### 4.4. Plasmids and Constructs: 

For the mammalian two hybrid assay, the CheckMate^TM^ Mammalian Two-Hybrid System (Promega, Madison, WI, USA) was used. The pACT-ERα-LBD construct was generated by cloning the LBD of ERα into the pACT vector to produce the ERα-LBD protein fused to the VP16 activation domain. The SRC-3 coactivator peptide (aa 614–698) containing LXXLL (L, leucine; X, any amino acid) motifs 1 and 2 was cloned into the pBIND vector to generate the pBIND-SRC-3 construct expressing the SRC-3 protein fused to the GAL4-DNA binding domain. Full-length human ERα (hERαWT) was encoded on a pcDNA3.1 expression plasmid. Point mutations in the LBD were generated with the QuikChange mutagenesis kit (Stratagene, San Diego, CA, USA) using the hERαWT template. Mutagenic primers were generated using a primer design tool (Agilent, Santa Clara, CA, USA). The pGL2.TATA.Inr.luc plasmid (Addgene, Cambridge, MA, USA, plasmid 11,354) was used as the estrogen-responsive luciferase reporter expression plasmid. AR, GR and PR were expressed from pcDNA3.1, pGR and pSG5-PRB mammalian expression vectors respectively as described previously [39].

### 4.5. Luciferase Transcriptional Assay

This assay was performed as described previously [24]. Briefly, ERα-positive T47DKBluc cells were grown in phenol red-free RPMI 1640 supplemented with 10% CSS for 5 days. The cells were seeded on 96-well plates (2 × 10^4^ cells/well). After 24 h, the cells were treated with test compounds in the presence of 1 nM E2. Twenty four hours post-treatment, the cells were lysed with 50 μL of 1× passive lysis buffer (Promega, Madison, WI, USA). Twenty microliters of the lysate from each treatment were transferred onto a white, 96-well, flat-bottomed plate (Corning Life Sciences, Corelle, NY, USA), and the luminescent signal was measured after adding 50 μL of the luciferase assay reagent (Promega, Madison, WI, USA) on a Tecan M200Pro microplate reader (Tecan, Menedorf, Switzerland). Differences in growth were normalised against total protein concentration, which was measured by the bicinchoninic acid (BCA) assay.

### 4.6. TR-FRET Assay

The LanthaScreen TR-FRET ER Alpha Coactivator Assay kit (PV4544; Life Technologies) was used as per the manufacturer’s instructions and described previously [24]. To assess the binding of VPC-16464 to the AF2 site, it was incubated with GST-tagged ERα-LBD (final concentration, 7.25 nM) in the presence of a fluorescently-labelled co-activator peptide (fluorescein-PGC-1α) at a final concentration of 250 nM, terbium (Tb)-labelled anti-GST antibody (final concentration, 5 nM) and E2 (final concentration, EC_80_ 6.1 µM). Cold SRC-2–3 and PGC-1α peptides were used as positive controls for AF2 site-specific binding. TR-FRET was analysed on a Synergy 4 hybrid microplate reader (BioTek, Winooski, VT, USA) with the settings at 340-nm excitation and 495- and 520-nm emission. The emission ratio (520:495) was analysed and plotted.

### 4.7. E2 Displacement Assay

E2 displacement was assessed with the PolarScreen ERα Competitor Assay Green kit (P2698; LifeTechnologies) as per the instructions of the manufacturer. Briefly, 50 µL of assay buffer containing VPC-16606 were added to a 50-μL mixture containing 2× full-length ERα and flourescein-E2 in each well of a 386-well black plate to obtain final concentrations of 0.2–6.3 μM of the test compound in the presence of 25 nM full-length ERα and 4.5 nM flourescein-E2. After 2 h, polarisation was measured on a Tecan F500 microplate reader.

### 4.8. BLI Assay

The direct binding of small molecules to the ERα-LBD was quantified by BLI using an Octet RED apparatus (Pall ForteBio, Menlo Park, CA, USA). ERα LBD (amino acids 302–552) was cloned into the pAN4 AviTag™ Vector, (Avidity, Aurora, CO, USA). Biotinylated ERα-LBD was purified as described previously [24]. Biotinylated protein (bERα LBD at 0.05 mg/mL) was bound to the super streptavidin sensors (Pall ForteBio) overnight at 4 °C in assay buffer (20 mM Tris, pH 7.5, 500 mM NaCl, 0.2 mM tris(2-carboxyethyl)phosphine TCEP, 0.02 mM E2, 5% glycerol and 5% dimethyl sulfoxide (DMSO). The compounds were dissolved in the assay buffer in a two-fold dilution series ranging from 3.1–100 μM. In all experiments, a known AF2-interacting peptide, PGC-1α (Elim Biopharmaceuticals, Hayward, CA, USA) was used as a control to confirm the functionality of the bERα LBD.

### 4.9. Cell Viability Assay

Cell viability was determined using the Presto Blue cell viability assay (Thermo Fisher Scientific, Waltham, MA, USA). Cells were seeded in clear bottom black-walled 96-well plates at a density of 5 × 10^3^ cells/well in 100 μL of their respective media. On the following day, the cells were treated with test compounds (0.001–22.5 μM) in the presence of 1 nM E2 and incubated at 37 °C. After 96 h, 30 μL of Presto Blue reagent was added and incubated for 10 min at 37 °C. The fluorescent signal from viable cells was measured at 590 nm.

### 4.10. qRT-PCR

mRNA levels were analyzed after treatment of MCF7 and TamR3 cells for 24 h with the test compounds as described previously [24]. The fold change in expression of the genes was calculated using the 2^−ΔΔCt^ method with *GAPDH* as the internal control.

### 4.11. Mammalian Two Hybrid Assay

MDA-MB-231 cells were seeded on 96-well plates (2 × 10^4^ cells/well) in 150 µL of medium. The cells were co-transfected with 25 ng each of pACT-ERα-LBD, pBIND-SRC-3, pG5luc and a constitutively-active Renilla reporter plasmid using TransIT-2020 reagent (Mirus, Madison, WI, USA). Cells were treated next day with the test compounds in the presence of 1 nM E2. After 24 h, the cells were lysed and luminescence was measured as for the luciferase transcriptional assay described above.

### 4.12. Western Blotting

Serum-starved MCF7 and TamR3 cells were seeded onto a six-well plate at a density of 6 × 10^5^ cells/well and treated the following day with VPC-16606 in the presence of 1 nM E2. One micromolar OHT was used as the positive control. After 24 h, cells were lysed in 1× radioimmunoprecipitation assay (RIPA) buffer. Twenty five micrograms of protein were loaded into SDS-PAGE gels and transferred to PVDF membranes. Membranes were incubated with pS2, PR Cyclin D1 and CDC2 antibodies or control α-actin antibody. Bound antibodies were detected using HRP-conjugated secondary antibodies. Chemiluminescence was detected with an Amersham ECL detection kit (GE Healthcare Life Sciences, Chicago, IL, USA), and bands were visualised using the G:BOX imager (Syngene, Cambridge, UK).

### 4.13. Chromatin Immunoprecipitation (ChIP)

E2-deprived MCF7 cells (1 × 10^7^ cells per 10 cm plate) were treated for 24 h with DMSO alone, DMSO + E2 or compound + E2. One percent formaldehyde was added for 10 min at room temperature to perform DNA-protein crosslinking and quenched by treatment with 125 mM glycine for 5 min. Cell lysates sonicated with Sonic Dismembrator 550 instrument (Thermo Fisher Scientific, Waltham, MA, USA) to yield DNA fragments of 200–1000 bp in size. Lysates (from 3.3 × 10^6^ cells) were immunoprecipitated with 5 g of anti-ERα antibody or 1 g of rabbit isotype control IgG (Santa Cruz Biotechnology, Dallas, TX, USA) using the EZ-ChIP chromatin immunoprecipitation kit (Millipore, Billerica, MA, USA). Bound DNA was quantified by qPCR (SYBR Green master mix, Invitrogen, Carlsbad, CA, USA) using the following primer sets: *pS2* enhancer, forward 5′-GTACACGGAGGCCCAGACAGA-3′ and reverse 5′-AGGGCGTGACACCAGGAAA-3′; *GAPDH* promoter, forward 5′-TACTAGCGGTTTTACGGGCG-3′ and reverse 5′-TCGAACAGGAGCAGA GAGCGA-3′. The qPCR results are presented as fold enrichment of PCR amplification over control IgG antibody and normalized based on the total input (no precipitated chromatin). Primers for the *GAPDH* promoter were used as a negative control.

### 4.14. Statistical Analysis

Data were analyzed and dose response curves generated using GraphPad Prism 6. Unpaired, two-tailed, Student’s *t*-tests were performed to analyze statistical significance between groups in data represented as bar graphs using GraphPad Prism 6 software (GraphPad Software, San Diego, CA, USA).

## 5. Conclusions

In summary, a series of benzothiophenone derivatives was designed and evaluated for their ability to inhibit human ERα, a primary drug target in the majority of BCa cases. Unlike conventional SERMs, these compounds bind to an alternative target site on ERα called the AF2 pocket and exhibit a novel mechanism for inhibition of the receptor. The most important feature of the developed AF2 inhibitor is its significant inhibitory activity against constitutively-active and clinically-relevant mutant forms of ERα. The results of this study suggest that blocking ERα action using AF2 inhibitors can provide a viable alternative (or complimentary) approach to existing endocrine therapies.

## Supplementary Materials

Supplementary materials can be found at http://www.mdpi.com/1422-0067/19/2/579/s1.

## Abbreviations

ER-α	Estrogen receptor-α
AF2	Activation function-2
BCa	Breast cancer
LBD	Ligand binding domain
MD	Molecular dynamics
BLI	Biolayer interferometry
