# Supplementary material for: Benzothiophenone Derivatives Targeting Mutant Forms of Estrogen Receptor-α in Hormone-Resistant Breast Cancers
Source: Int J Mol Sci. 2018 Feb 15;19(2):579. doi: 10.3390/ijms19020579 (PMC5855801; doi:10.3390/ijms19020579)
Supplement: Supplementary file 1 [file ijms-19-00579-s001.pdf]

Supplementary Materials: Benzothiophenone Derivatives Targeting Mutant Forms of Estrogen Receptor-α in Hormone-Resistant Breast Cancers

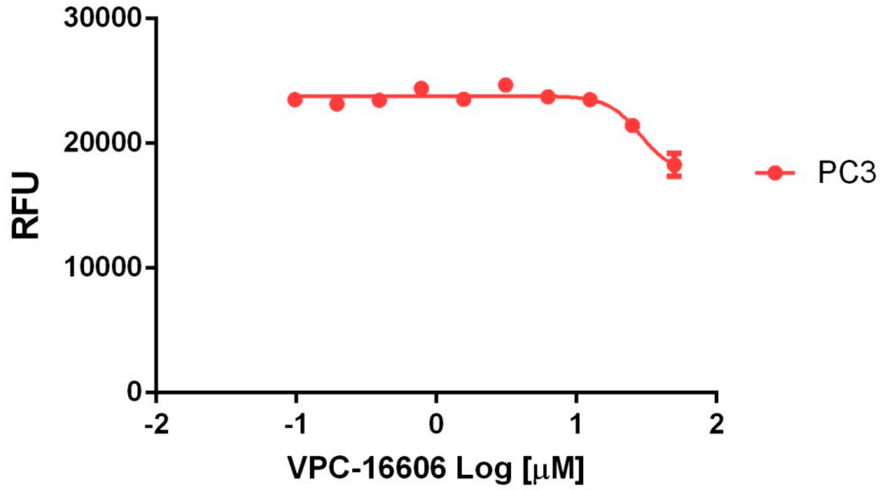

Figure S1. Effect of VPC-16606 on viability of PC3 cells as measured in the Presto Blue assay. Cells were treated for 96 hours with 2-fold dilution of the compound starting at 50μM. Error bars represent standard deviation for two independent experiments performed in triplicates.

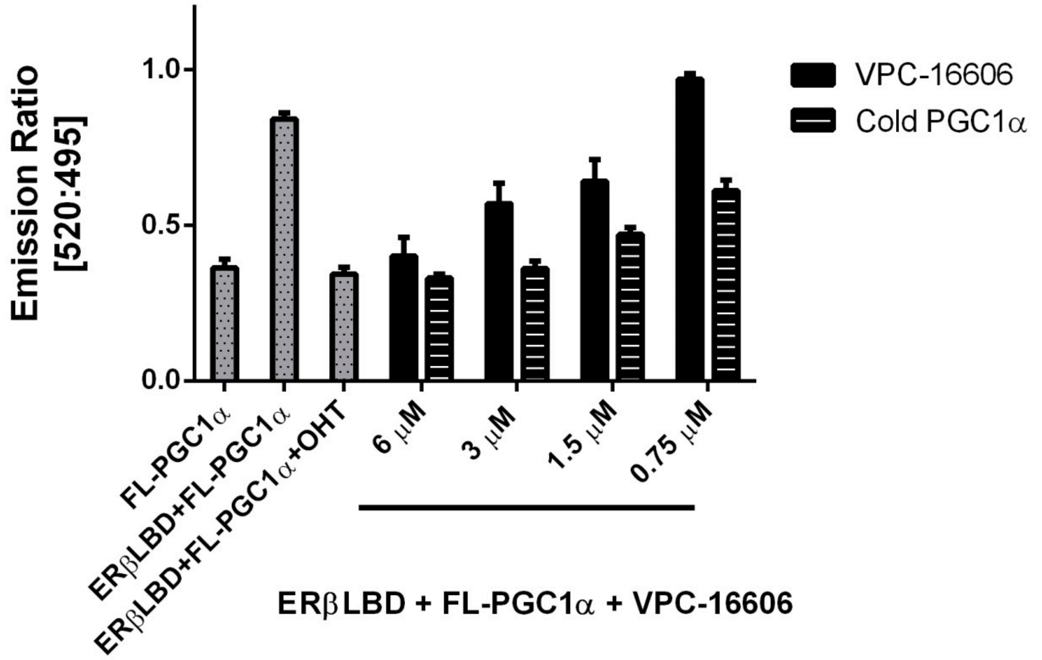

Figure S2. TR-FRET Assay showing displacement of Fluorescein labelled PGC1α peptide by VPC-16606 upon incubation with ERβ-LBD. OHT was used at 1μM. Error bars represent standard deviation for two independent experiments performed in triplicates.
